# Safety and Effectiveness of Thiopurines and Small Molecules in Elderly Patients with Inflammatory Bowel Diseases

**DOI:** 10.3390/jcm13164678

**Published:** 2024-08-09

**Authors:** Aleksandra Strigáč, Miłosz Caban, Ewa Małecka-Wojciesko, Renata Talar-Wojnarowska

**Affiliations:** 1 Department of Digestive Tract Diseases, Faculty of Medicine, Medical University of Lodz, 90-153 Lodz, Poland; milosz.caban@stud.umed.lodz.pl (M.C.); ewa.malecka-panas@umed.lodz.pl (E.M.-W.); renata.talar-wojnarowska@umed.lodz.pl (R.T.-W.); 2 Department of Biochemistry, Faculty of Medicine, Medical University of Lodz, 92-215 Lodz, Poland

**Keywords:** Crohn’s disease, inflammatory bowel diseases, JAK inhibitors, S1PR modulators, thiopurines, toxicity, ulcerative colitis

## Abstract

The management of inflammatory bowel diseases (IBD) requires weighing an individual patient’s therapeutic benefits and therapy-related complication risks. The immunomodulators that have been commonly used so far in IBD therapy are thiopurines, including 6-mercaptopurine and azathioprine. As our understanding of the IBD pathomechanisms is widening, new therapeutic approaches are being introduced, including the Janus kinase (JAK) inhibitors and Sphingosine 1-phosphate receptor (S1PR) modulators’ development. Non-selective JAK inhibitors are represented by tofacitinib, while selective JAK inhibitors comprise filgotinib and upadacitinib. As for the S1PR modulators, ozanimod and etrasimod are approved for UC therapy. The number of elderly patients with IBD is growing; therefore, this review aimed to evaluate the effectiveness and safety of the oral immunomodulators among the subjects aged ≥60. Possible complications limit the use of thiopurines in senior patients. Likewise, the promising effectiveness of new drugs in IBD therapy in those with additional risk factors might be confined by the risk of serious adverse events. However, the data regarding this issue are limited.

## 1. Introduction

Inflammatory bowel diseases (IBD), represented primarily by Crohn’s disease (CD) and ulcerative colitis (UC), are chronic immune-mediated disorders characterised by a progressive and unpredictable course, as well as an increasing global incidence over the last few decades [1,2]. There are two peak ages of the incidence of IBD—the first one is at 20–40 years old, while the other one, associated with a constantly growing number of new diagnoses, is observed at 60–70 years old [3]. The most recent data suggest that the IBD prevalence is expected to increase two-fold by the year 2030 compared to the year 2010 and to affect 1% of the Western population, accounting for ten million cases [4]. It is estimated that approximately 15% of new IBD cases are diagnosed among elderly patients. In addition, about 30% of all subjects with IBD are over 60 years old [3,5]. It must be emphasised that due to the ageing of the general population, improvement in life expectancy, increase in the incidence of IBD, and the existence of the second disease peak, especially in UC, the population of elderly patients with IBD will be more numerous and will become increasingly important in the therapeutic management of IBD.

The elderly onset IBD is characterised by a distinct clinical presentation, particular disease phenotype, and unique natural history [3,6]. The biological changes related to ageing, such as immunosenescence, alterations in the composition of gut microbiota, frailty, and decreased homeostatic reserves strongly affect the therapeutic effects of drugs [7]. Moreover, the significantly higher frequency of comorbidities in the elderly compared to that in younger patients contributes to an elevated risk of drug interactions, polypharmacy, severe infections, and malignancy [8,9,10]. Unfortunately, elderly patients are not commonly included in medical trials due to restrictive eligibility criteria. Therefore, a limited amount of data from randomised clinical trials are available. These factors should be considered while personalising therapeutic strategies for patients with IBD over 60 years old. Hence, to gain a clearer understanding of the utility of oral immunomodulators, including thiopurines and small molecules, in treating elderly patients with IBD, this review discusses their use in those subjects on the basis of the most recent scientific evidence.

## 2. Search Strategy

PubMed, Google Scholar, Wiley, Springer, Scopus, Embase, and Web of Science databases were systematically and extensively searched for the collected bibliography. The search included all the studies published until May 2024, using the following keywords alone or in combinations: inflammatory bowel diseases, Crohn’s disease, ulcerative colitis, thiopurines, azathioprine, 6-mercaptopurine, elderly, therapy, effectiveness, safety, JAK inhibitors, S1PR modulator, ozanimod, etrasimod, upadacitinib, tofacitinib, and filgotinib. Articles that concerned biological therapy were excluded. Clinical trials were searched using the ClinicalTrials.gov database. The searches were filtered to include only those studies published in English.

## 3. Thiopurines

**Figure 1:**
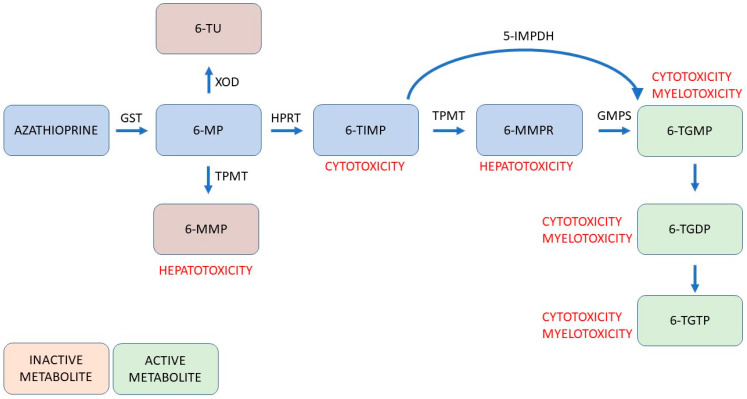
Schematic view of simplified metabolism of thiopurines considering the properties of individual metabolites and their potential toxic influence. Abbreviations: 5-IMPDH, 5-inosine monophosphate dehydrogenase; 6-MMP, 6-methylmeracptopurine; 6-MMPR, 6-methylmeracptopurine ribonucleotide; 6-MP, 6-mercaptopurine; 6-TIMP, 6-thioinosine monophosphate; 6-TGDP, 6-thioguanosine diphosphate; 6-TGMP, 6-thioguanosine monophosphate; 6-TGTP, 6-thioguanosine triphosphate; 6-TU, 6-thiouric acid; GMPS, guanosine monophosphate synthetase; GST, glutathione S-transferase; HPRT, hypoxanthine-guanine phosphoribosyltransferase; TPMT, thiopurine methyltransferase; XOD, xanthine oxidase [13].

Thiopurines, represented by 6-mercaptopurine (6-MP) and azathioprine (AZA), are purine antimetabolites used in the maintenance therapy of CD and moderate to severe UC [11]. They interfere with cellular proliferation and, as a result, modulate innate and adaptive immunity [12]. After oral administration, they undergo intracellular activation and are metabolised with enzymes via various pathways into active and inactive metabolites (Figure 1). Unfortunately, some of the metabolites are responsible for possible adverse events and drug interactions (mainly with warfarin, mesalazine, furosemide, and allopurinol).

The most common side effects of the treatment are leukopenia, myelosuppression, hepatotoxicity, kidney injury, gastric intolerance, and pancreatitis. In addition, an increased risk of non-melanoma skin cancer (NMSC), lymphoma, and cervical cancer is observed in patients exposed to long-term therapy with thiopurines. Calafat et al. [14] confirmed the statistically significant increased risk of thiopurine-related adverse events in the elderly patients with IBD who started the therapy after they turned 60, compared with the subjects who started the therapy earlier (43.4 vs. 29.7%; *p* < 0.01). These events included infections (3.6 vs. 2.0%; *p* < 0.001), neoplasms (1.5 vs. 0.2%; *p* < 0.001), myelotoxicity (14 vs. 7.6%, *p* < 0.001), anaemia (4.3 vs. 1.2%; *p* < 0.001), leukopenia (10.4 vs. 6.1%; *p* < 0.001), hepatotoxicity (9 vs. 4.7%, *p* < 0.001), and digestive intolerance (12.3 vs. 10%; *p* = 0.002). In addition, the elevated risk of malignancy and mortality was confirmed in the patients with IBD aged ≥65 treated with thiopurines, in comparison with the ones treated with anti-tumour necrosis factor-alpha (anti-TNF-α) antibody (HR = 3.017; 95% CI: 1.050–8.666; *p* = 0.0403, and HR= 3.682; 95% CI: 1.192–11.377; *p* = 0.0235, respectively) [15]. Interestingly, the administration of thiopurines in elderly subjects with IBD is also associated with an elevated independent risk of pancreatic cancer (SIR 7.29, 95% CI 1.82–29.16) [16], acute myeloid leukaemia/myelodysplastic syndrome (adjusted HR 3.05; 95% CI 1.54–6.06, *p* = 0.0014 for actual use of thiopurines with a cumulative exposure of <2 years and adjusted HR 2.32; 95% CI 1.22–4.41, *p* = 0.0101 for actual use of thiopurines with a cumulative exposure of ≥2 years) [17], as well as kidney and urinary bladder cancers, primarily in male cases [18]. Generally, it is estimated that thiopurine therapy discontinuation due to adverse events occurs in almost one-third of patients, indicating unsatisfactory tolerance of the treatment [19,20]. A retrospective study performed by Suárez Ferrer et al. [21] among patients with CD aged 17 to 84 showed thiopurine failure in approximately 38% of cases due to adverse events or a lack of effectiveness. In turn, Jorissen et al. [22] revealed that withdrawal of thiopurine in elderly patients with IBD who achieved clinical or endoscopic remission was associated with a relapse of the disease after a median follow-up of 66 months in 30.8% of subjects. It is worth emphasising that the median therapy duration in those experiencing a relapse was significantly shorter compared with the patients who remained in remission (median 45 vs. 103 months, *p* = 0.005). Moreover, cancer developed in 26 (18.3%) out of 142 subjects. This indicates that these patients require a close follow-up during and after the therapy and the decision to use thiopurines should be prudent and personalised.

Regretfully, no controlled or retrospective studies addressing the effectiveness of thiopurines in elderly patients have been conducted, and the literature on this issue is insufficient. A meta-analysis in 2020 demonstrated that the overall exposure to immunomodulators in the elderly onset CD and UC was significantly lower than in the patients with the adult onset of the disease [23]. Surprisingly, in a retrospective cohort study on the association between medication and life expectancy in seniors with IBD, Kuenzig et al. [24] reported that the monotherapy with an immunomodulator, in comparison with the combination therapy with a biologic, decreased life expectancy by 5.1 (95% CI 2.3–7.8) and 2.8 years (95% CI 0.1–5.5) in elderly females and males with IBD, respectively.

Nevertheless, the literature review revealed undisputable advantages of the thiopurines therapy, among them the oral route of administration and low cost of the treatment [25]. Additionally, it was proven that the combined administration of azathioprine and infliximab, the primary representative of anti-TNF-α antibodies, increases the effectiveness of the anti-TNF-α therapy, probably via the inhibition of anti-drug antibody formation. This phenomenon contributes to azathioprine’s usefulness in maintaining long-term remission in patients who tolerate the therapy well [26]. What is more, the early introduction of thiopurines with anti-TNF-α therapy accelerates the induction of steroid-free remission [12]. Moreover, thiopurines efficiently manage the perianal CD and contribute to the reduction in the frequency of perianal surgery. Furthermore, the 20-year-long national population-based cohort study showed that thiopurine use for longer than 12 months was associated with a 70% reduction in the risk of colectomy in patients with elderly onset UC. However, this effect was not observed in CD [27].

Interestingly, it seems that thiopurines may reduce the risk of venous thromboembolism that is elevated at the baseline in elderly patients, especially those with IBD [28]. Moreover, the therapy with 6-mercaptopurine may significantly increase the chance of mucosal healing in some subjects. Surprisingly, the post hoc analysis of the TOPPIC study revealed that 6-mercaptopurine increases the likelihood of complete mucosal remission only in smoking patients [29].

Azathioprine and 6-mercaptopurine both might be the first thiopurine prescribed. Moreover, they might be used interchangeably in case of intolerance of the treatment with the initial thiopurine. Adjusting the dose is crucial when switching drugs [30]. In the nationwide, observational, retrospective Spanish study based on the ENEIDA registry regarding the switching to a second thiopurine, 173 (13.5%) patients started the first thiopurine at over 60 years of age—in 164 (94.8%), azathioprine was initially introduced. The median time of exposure to the first thiopurine was significantly shorter among the elderly compared with adult patients—in 142 (88.2%) elderly subjects, the switching to the second thiopurine was required earlier than 6 months from the beginning of the therapy. It is noteworthy that the group of elderly patients had a significantly higher proportion of cases in whom the first thiopurine was discontinued due to hepatotoxicity (40 (23.1%) vs. 129 (11.7%) subjects) or myelotoxicity (19 (11.0%) vs. 63 (5.7%) patients) and a lower proportion of discontinuation due to gastrointestinal toxicity (87 (50.3%) vs. 676 (61.2%) cases), as compared with young adults. Starting thiopurines at over 60 years of age, intolerance to 6-mercaptopurine as the first thiopurine, time of exposure to the first thiopurine <6 months, and acute pancreatitis induced by the first thiopurine were the risk factors for developing intolerance to the second thiopurine [31].

Interestingly, in 2012, an analysis was carried out by Lipka et al. [32], regarding the side effect profile of 6-mercaptopurine/azathioprine in elderly patients with UC or CD among 41 subjects characterised by the mean age of 70.4 years and average duration of the therapy equal to 48.5 months. Here, 30 (73.2%) patients experienced one side effect, whereas 8 (19.5%) had multiple occurrences. The adverse events included leukopenia, pancytopenia, infections, rash, and elevated liver enzymes. Malignancies had developed in two (4.9%) patients and included skin cancer, bladder cancer, and prostate cancer. The discontinuation of the treatment due to the severity of adverse events was required in four (9.8%) cases—the rest of the subjects continued the therapy at the current or lowered dosage owing to its beneficial influence on the activity of IBD.

Although thiopurines remain a reliable therapeutic option for the treatment of IBD, their use in elderly patients should be personalised. This ought to be performed on the basis of the evaluation of potential benefits and contraindications for the therapy, for example, the existence of concomitant diseases. Generally, age above 65 years is not a contraindication for thiopurines. However, a detailed clinical assessment is crucial, and the initiation of the therapy in the elderly should be carefully considered, as the risk of adverse events, including infections and malignancies, is noticeably increased among them [33]. The monitoring of potential side effects during the therapy is essential. All patients treated with thiopurines should be annually followed up by a dermatologist. In addition, women should participate in cervical cancer screening programs. The therapy with thiopurines requires regular monitoring of peripheral blood count, aminotransferases activity, and kidney function [13]. Also, in elderly subjects, particular caution should be exercised when thiopurines are administered concurrently with immunosuppressants with a different mechanism of action.

Taking everything into consideration, it seems that the use of thiopurines in older patients should be secondary to other efficient, safer drugs, especially in case of comorbidities increasing the risk of infections or cancer diseases. Some authors even advocate for the withdrawal of thiopurines in patients aged ≥60 years due to possible life-threatening complications [33]. Notably, it is postulated that the combination of thiopurines with anti-TNF-α inhibitors should not be applied in elderly patients [27].

Due to the growing number of patients that become irresponsive to or do not tolerate conventional therapy, including thiopurines, there is a constant need for new drugs that are both effective in the maintenance of IBD and are encumbered with a low risk of adverse events. In the last few years, thanks to the broader understanding of the underlying disease processes, several novel small-molecule drugs have been developed and approved for the treatment of IBD. These include two groups of medicaments: inhibitors of the Janus kinase Signal Transducer and Activator of Transcription (JAK-STAT) signalling pathway and Sphingosine 1-phosphate receptor (S1PR) modulators.

## 4. JAK Inhibitors

**Figure 2:**
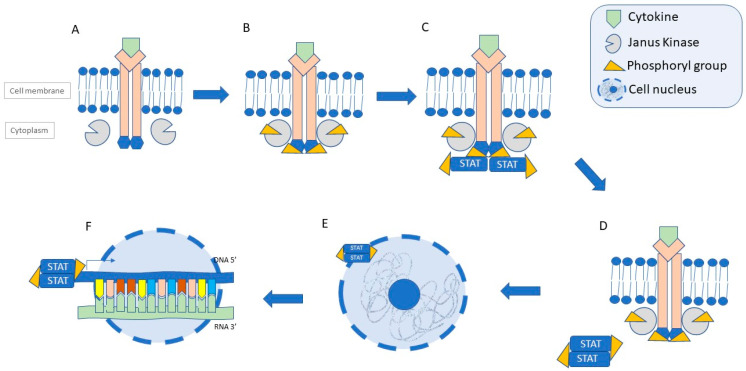
Schematic view of the JAK-STAT signalling pathway. (**A**) Recruitment of JAK molecules by activated cytokine receptor. (**B**) Phosphorylation of JAK molecules and receptor domains. (**C**) Recruitment of STATs and their phosphorylation. (**D**) Dissociation from the receptor and dimerization of STATs. (**E**) Migration of STAT dimers into the nucleus. (**F**) Transcription of pro-inflammatory genes. Abbreviations: DNA—deoxyribonucleic acid; RNA—ribonucleic acid; STAT—signal transcuder and activator of transcription [36].

Janus kinase (JAK) refers to a group of non-receptor intracellular tyrosine kinases. It comprises four proteins: JAK1, JAK2, JAK3, and TYK2, which are involved in signalling for specific extracellular cytokines, such as interleukins (IL, e.g., IL-2, IL-4, IL-7, IL-9, IL-15, and IL-21) and interferon-gamma (IFN-γ). Through phosphorylation, they activate the STAT proteins, which are then translocated to the cellular nucleus, where the transcription of the pro-inflammatory genes is induced (Figure 2) [34]. It is well known that JAK1 is responsible for the cytokine-mediated inflammatory response, JAK2 mediates myelopoiesis and erythropoiesis, while JAK3 is involved in lymphopoiesis. In turn, Tyk2 significantly affects the transduction of IL-6, IL-10, IL-12, IL-23, IL-10, and INF signalling. The dysregulation of the JAK pathway is one of the mechanisms involved in the pathogenesis of CD and UC. Consequently, targeting the JAK pathway presents a promising therapeutic approach in patients with IBD [35].

JAK inhibitors that have been approved so far for the treatment of IBD can be divided into two selectivity classes due to their target kinases. The non-selective drug is tofacitinib—the inhibitor characterised by the highest activity against JAK3. The second group consists of upadacitinib and filgotinib—both selective for JAK1. The varying selectivity between the two classes is linked to the differences in safety and effectiveness [34]. Generally, higher selectivity tends to result in fewer adverse events [37].

Due to their mechanism of action, adverse events in this group of drugs include suppression of haematopoiesis, severe infections of various aetiologies, and hypercholesterolemia. Furthermore, the dampening of NK cells and interferons, which are instrumental in tumour surveillance, might increase the risk of malignancies, but further research in this area is required [38]. Noteworthy, in 2021, the FDA alerted the public to the heightened risk of severe heart-related incidents, blood clot formation, and death linked to the use of JAK inhibitors, including tofacitinib, upadacitinib, and baricitinib (a JAK1 and JAK2 selective inhibitor approved for the therapy of rheumatoid arthritis (RA)), based on the outcomes of the ORAL clinical trial of tofacitinib in patients with RA. The hazard was even higher in patients aged ≥65 years, smokers, and those with pre-existing cardiovascular risk factors [39,40,41].

On the contrary, the systematic review and meta-analysis performed by Li et al. [42] did not reveal a significant difference in cardiovascular disease risk between patients with RA who received JAK inhibitors and those who received a placebo or other active agents. Nevertheless, risk assessment for a major adverse cardiovascular event (MACE) and venous thromboembolism (VTE) must be taken into consideration before prescribing JAK inhibitors, especially since patients with IBD already have an increased cardiovascular risk [43]. Moreover, various clinical factors contribute to an elevated risk of venous thromboembolism in patients with IBD. These factors include an active and extensive disease, a recent surgery (especially colorectal procedures), hospitalisation, pregnancy, and the use of corticosteroids. While younger age may be associated with a higher relative risk of VTE among patients with IBD, older individuals tend to experience a higher incidence of VTE and are more likely to present VTE events [27].

Among anti-inflammatory medications, JAK inhibitors carry one of the highest risks of infections, second only to anti-TNF-α inhibitors. Reactivation of Herpes zoster infection occurs frequently with all licensed JAK inhibitors, particularly in patients receiving high doses. It is important to note that patients with IBD are inherently at a higher risk for Herpes zoster compared to non-IBD individuals. Age and immunosuppression (e.g., steroid therapy) further elevate this risk. Owing to this fact, all patients should be screened for potential sources of infection as a part of qualification for therapy. Moreover, all eligible patients should be vaccinated [43].

Before the beginning of the therapy, during the initial treatment phase and at regular intervals afterwards, cholesterol levels should be assessed. Various studies highlight the increased prevalence of hyperlipidaemia in the elderly due to multiple factors, such as age-associated loss of hepatic LDL receptors, higher body mass index, larger waist circumference, and lower sex hormone levels [44,45,46]. Patients should receive appropriate interventions if cholesterol levels are elevated [43]. Gilroy and Wilson [47] observed statistically significant cholesterol level increases in a cohort of several dozen adults treated with JAK inhibitors for UC and CD. It was noticed that upadacitinib increased average cholesterol levels the most, followed by tofacitinib. The influence on the lipid profile in the filgotinib group was present but it was not as marked as in the previously mentioned groups of patients.

On the other hand, the characteristics of JAK inhibitors make them appealing, compared to monoclonal antibodies. These properties include oral administration, a short plasma half-life, lack of immunogenicity, and predictable pharmacokinetics. Due to their ability to quickly enter the bloodstream, JAK inhibitors exhibit a rapid onset of action and can provide swift relief from symptoms [37,48]. A meta-analysis including 66,159 patients with immune-mediated diseases proved that the mortality among those treated with a JAK inhibitor was comparable to that in a group of patients receiving a placebo (relative risk: 0.72, 95% CI 0.40–1.28). The overall mortality rate for individuals exposed to JAK inhibitors was 0.37 per 100 person-years [49]. This is important, as the chance of death rises exponentially with age, and multiple studies confirmed the association between higher mortality risk and inflammatory bowel diseases [35,38].

### 4.1. Tofacitinib

Tofacitinib was the first JAK inhibitor approved by the FDA for the treatment of UC. It inhibits JAK3 and JAK1 and, to a lesser extent, JAK2, with nearly no effect on TYK2. The daily dose in the induction phase is 10 mg twice daily (BID), and 5 mg twice daily during maintenance. As tofacitinib is mainly metabolised and eliminated by the liver, the dosage should be reduced by half in patients with moderate liver disease. Patients with severe liver impairment should not be treated with this JAK inhibitor. Moreover, small increases in creatinine have been observed with tofacitinib; therefore, caution is required in those with severe kidney disease [43,50]. Tofacitinib is metabolised by CYP3A4. Consequently, coadministration with different substrates, such as ketoconazole, tacrolimus, or cyklosporine, should be cautiously approached [50].

Regarding the effectiveness of tofacitinib in the elderly patients with UC, the meta-analysis presented by Lichtenstein et al. [51] showed some statistically significant differences in the induction phase between the groups of patients aged ≥60 receiving tofacitinib 10 mg BID and a placebo. The clinical response was seen in 64.2% versus 43.8%, respectively. Clinical remission was achieved in 22.0% of patients receiving tofacitinib versus 6.3% of those on a placebo, and endoscopic improvement was noticed in 35.8% of patients versus 18.8%, respectively. In the maintenance cohort, two doses of tofacitinib were administered—10 mg BID and 5 mg BID. Clinical response was accomplished in 71.0% and 72.7% of patients, respectively, compared to 10.3% in the placebo group. Clinical remission was observed in 54.8% of patients treated with a higher dose, in 59.1% of those with tofacitinib 5 mg BID, and in 3.4% of those receiving a placebo. Endoscopic improvement was confirmed in 61.3% and 59.1%, respectively, compared to 3.4% of patients on placebo.

As for the safety of the therapy, it was emphasised that the incident rates of adverse events and serious adverse events during the maintenance period were comparable between the analysed groups, with no age-related dependency. Generally, the therapy was well tolerated by the patients, and the adverse events were uncommon. However, the incidence of Herpes zoster infection was higher in those above 50 years old treated with tofacitinib 10 mg twice daily when compared to the lower dose or a placebo. Overall, the frequency of serious infections remained relatively consistent across different age groups. Nevertheless, the incidence rates of opportunistic infections, Herpes zoster, malignancies (excluding NMSC), NMSC itself, and major adverse cardiovascular events all showed an upward trend with increasing age. Noteworthy, older patients are generally encumbered with a higher risk of the diseases mentioned above [51,52].

The effectiveness of the therapy in the group of patients ≥65 years old was also recently assessed by Khan et al. [53] in the meta-analysis of the 53 cases of elderly patients receiving tofacitinib. After one year, 62.0% of patients were still on the drug, with effectiveness seen in 50.94%. The reasons for discontinuing the therapy included no response or a loss of response. Apart from two cases of Herpes zoster infections, no other adverse events were detected.

Another remarkable single-centre retrospective study was carried out by Viola et al. [54], during which the data of 30 patients aged 51 to 85, with a Charlson Comorbidity Index ≥2, treated with tofacitinib for UC were collected and analysed. Fourteen (46.7%) subjects were treated with the halved induction dose due to the individual risk factors, such as tobacco addiction, frailty, and increased cardiovascular or thrombotic risk. After one year of the treatment, steroid-free remission was achieved in 16 (53.3% of the initial number of participants aged ≥50) out of 19 (63.3% of the initial number of participants aged ≥50) patients who continued the therapy. As for the adverse events that led to the precocious ending of the treatment, two cases of malignancies (lymphoproliferative disorder and colorectal cancer) were recorded and one exacerbation of arthralgias. No thrombosis, MACE, or major infections were reported. It ought to be emphasised that 12 (40.0%) patients were already treated with anti-thrombotic agents due to comorbidities. Apart from that, two cases of benign-course Herpes zoster, which required temporary dose reduction, were observed. According to the review article published by Núńez et al. [35], the cases of Herpes zoster infections reported during different clinical trials of tofacitinib were mainly non-complicated and did not lead to the cessation of the treatment.

The results concerning the safety of the therapy were confirmed in yet another analysis of multiple retrospective randomised trials published by Shehab et al. [55]. In those with UC treated with tofacitinib, the risk of MACE was comparable to that in the patients receiving a placebo in both the induction and maintenance phases (OR 1.30, 95% CI 0.15–11.21 for tofacitinib). Unfortunately, three clinical trials evaluating the effectiveness of tofacitinib in patients with CD showed no significant impact on the course of the disease, neither in the induction nor the maintenance phase [37,56,57].

### 4.2. Filgotinib

Filgotinib is the JAK1 selective oral inhibitor approved for the treatment of UC. It is involved in the IL-2, IL-4, IL-5, IL-6, and type I interferon signalling. A standard dose is 200 mg once daily, regardless of meals. A biologically active aminotriazolopyridine metabolite that contributes to the parent compound’s activity is formed through the metabolic changes [50]. A vast majority of the drug is eliminated in the urine, and some clinical trials showed that the exposure to filgotinib and its metabolites was increased in patients with moderate and severe renal impairment. Thus, the recommended dose for such patients is 100 mg per day. No data regarding end-stage renal disease or severe liver insufficiency are available [58]. The progressive reduction in functioning glomeruli begins after the age of 40, as renal blood flow depletes by roughly 1% per year [59]. Therefore, adjustment of the dose of filgotinib might be required in elderly patients.

Apart from renal or hepatic impairment, other factors that influence the choice of the therapy in a population of elderly patients are potential interactions with drugs commonly used by the infirm due to comorbidities. There is some inconsistency regarding filgotinib’s potential for interfering with concomitant drugs. In vitro studies showed that carboxylesterase 2—the main enzyme involved in the metabolism of filgotinib—might be inhibited by medicaments, such as fibrates, beta-blockers, or calcium channel blockers. However, the clinical significance of this finding is yet to be studied. Furthermore, filgotinib might increase the exposure to the OATP1B1 and OATP1B3 (hepatic uptake transporters) substrates, such as statins or sartans. Therefore, their coadministration is not recommended [58].

To assess the influence of ageing on filgotinib’s metabolism, Namour et al. [60] conducted an open-label phase I study among 30 healthy subjects divided equally into 3 groups according to the following ages: 40 to 50 years, 65 to 74 years, and ≥75 years old. It was noted that the elimination half-time of filgotinib and the excretion of filgotinib’s metabolite in the urine were comparable among all the groups. On the contrary, the elimination half-time of the filgotinib’s metabolite was subtly longer in the patients aged ≥65 than in those ≤50, with no apparent difference between the elderly groups. As for the safety of the therapy, no deaths or serious adverse events were observed. Two cases of moderate complications of filgotinib intake were noticed, being migraine and urinary continence. Generally, filgotinib at a daily dose of 100 mg was safe and well tolerated among the elderly patients.

The effectiveness and safety of the filgotinib therapy were assessed in a SELECTION and SELECTIONLTE phase 2b/3, double-blind, randomised, placebo-controlled trial. In the induction phase, clinical remission at week 10 among the 149 patients aged ≥ 60 in the treatment arm was achieved by 20 people (13.4%), in comparison to 3 (8.6%) out of 35 in the placebo arm. The partial Mayo Clinic Score remission (defined as pMCS ≤ 1) in these groups was observed in 56 (37.6%) patients and 6 (17.1%) patients, respectively. At week 58, amid 45 patients still receiving filgotinib, 24 (53.3%) were in pMCS remission versus 5 (18.5%) of those receiving a placebo.

As for the long-term extension study (LTE), at week 24, pMCS remission was recorded in 52 (35.9%) elderly patients. Among all 173 patients with UC aged ≥60 who were treated with filgotinib during both studies, 139 (80.3%) adverse events were observed, out of which 39 (22.5%) were classified as grade ≥3. Most commonly, infections were noticed (80 (46.2%) cases, 4 (2.3%) of which were serious). Other adverse events included malignancies (11 (6.4%) cases), stroke (1 (0.58%) case), 1 (0.58%) case of myocardial infarction, and 2 (1.2%) cardiovascular deaths; in these cases, the patients had additional cardiovascular risk factors. This outcome suggests that filgotinib might have an acceptable safety profile in elderly patients [61].

Similarly, Shimada et al. [62] presented the case of an 86-year-old male patient who developed a SARS-CoV-2 vaccination-related elderly onset UC. An initial improvement was achieved by the administration of high doses of prednisolone combined with 5-aminosalicylic acid. However, a relapse of the disease was observed due to the steroid tapering. Thus, filgotinib was introduced, and steroid-free remission was attained. No adverse events of the therapy with a JAK inhibitor in this patient were mentioned.

Of note is that the risk of Herpes zoster is associated with all JAK inhibitors. The established risk factors for Herpes infections include age ≥ 60, lower body mass, and prior TNFα inhibitors exposure. Nonetheless, the frequency of serious Herpes zoster infections in patients receiving filgotinib has been very low, regardless of the dose [35].

Disappointingly, the initial promising data regarding the mileage of filgotinib in the treatment of CD were not confirmed during further research. In the randomised, double-blind, placebo-controlled phase 2 FITZROY study, filgotinib administered at a daily dose of 200 mg resulted in clinical remission for 60 (46.8%) patients with moderate-to-severe CD, compared to 10 (22.7%) in the placebo group (*p* = 0.008). Notably, patients achieved clinical remission within just four weeks, whereas tofacitinib required a minimum of eight weeks to induce remission [38,63]. Unfortunately, in 2023, Galapagos—the biotech company that developed filgotinib—announced that during the phase 3 DIVERSE trial, neither of the cohorts met the co-primary endpoints of clinical remission and endoscopic response. Therefore, further progression of the drug in this disease has been suspended [64].

### 4.3. Upadacitinib

Upadacitinib is a JAK1 selective oral inhibitor approved for the treatment of both UC and CD [65]. It is available in an extended-release formulation and the therapy schedule is slightly different in both IBDs. The induction phase lasts 8 weeks, whereas in CD it is elongated to 12 weeks. In both diseases, the induction dose is 45 mg once daily and the maintenance dose is 15 mg once daily. In severe, refractory, or extensive courses, doubling of the duration of the induction phase or elevation of the daily maintenance dose to 30 mg might be taken into consideration.

Of note, according to the open-label, single-dose study conducted by Treuman at al. [66] among patients with mild and moderate hepatic impairment, the dose reduction of upadacitinib is not required. During the study, the drug in a daily dose of 15 mg was well tolerated by the subjects, and no serious adverse events were reported. Diarrhoea, headache, dizziness, and nausea were observed.

Another study, supervised by Mohamed et al. [67], showed that there is also no dose adjustment needed in patients with mild to severe renal impairment. Likewise, one case of mild diarrhoea and upper respiratory infection was reported, with no moderate or serious adverse events observed during the study.

Upadacitinib is mainly metabolised by CYP3A4, and an in vivo study demonstrated that strong CYP3A inhibition had a weak effect on upadacitinib exposure, while broad CYP induction resulted in a moderate effect [68]. The assessment of the drug–drug interaction (DDI) potential for upadacitinib as a perpetrator drug did not reveal significant concerns from a DDI perspective [69].

The data regarding the effectiveness and safety of upadacitinib among the elderly is quite limited so far. However, a multicentre retrospective cohort study, comprising 26 patients aged ≥60 (range 60–80 years old) with moderate to severe UC and previous history of corticosteroids and biological therapy, was conducted by Chowla et al. [70], and 21 (80.8%) patients reported clinical improvement. Out of those in whom the symptoms did not improve, three (11.5%) patients required colectomy due to refractory disease and one due to colon neoplasia. Adverse events, such as hyperlipidaemia, occurred in six (23.1%) patients, two (7.7%) suffered from acne or rash, and one (3.8%) developed severe oral ulcerations. Generally, the safety profile of upadacitinib in the elderly was comparable to that in the general population.

Furthermore, a case series report was published by Levine et al. [71], in which two patients aged ≥60 were mentioned. The first one, a 73-year-old female with left-sided UC previously treated with TNFα inhibitors and tofacitinib, received upadacitinib for 4 months. Clinical improvement was achieved, and no adverse events were observed during the treatment or the four-month follow-up. The second patient was a 60-year-old male with left-sided UC and a history of therapy with TNFα inhibitors and tofacitinib. Clinical improvement and normalisation of the calprotectin level were obtained during a 2.5-month treatment. An adverse event was recorded but, unfortunately, the severity of it remains unspecified.

The comparative multicentre cohort study proved that the general effectiveness of upadacitinib at achieving steroid-free clinical remission in UC was significantly higher than that of tofacitinib. The ratios of endoscopic response/remission were comparable [72].

The safety and effectiveness of upadacitinib in the therapy of CD were evaluated in the randomised, double-blind CELEST study among 220 adults aged 19 to 76. Regrettably, no analysis of the effectiveness in patients stratified by age is available. The safety outcomes were quite promising. Most of the adverse events were classified as mild or moderate. Most frequently, headache, worsening of symptoms, nausea, vomiting, acne, and urinary and upper respiratory infections were observed. In the elderly, a few cases of adverse events were reported, such as a malignant neoplasm of the thymus in a 62-year-old male and an acute myocardial infarction in a 67-year-old male, with at least 3 cardiovascular risk factors. No events of deep vein thrombosis or pulmonary embolism occurred [73]. Among all the patients receiving upadacitinib, regardless of their age, three cases of Herpes zoster were reported [35].

## 5. S1PR Modulators

**Figure 3:**
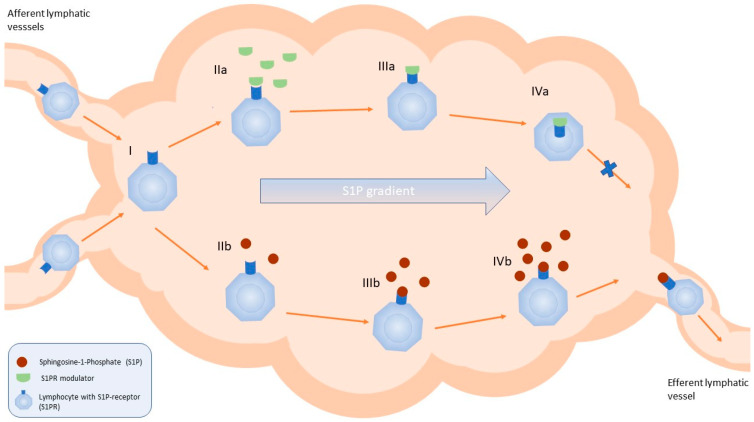
Schematic view of the simplified mechanism of action of S1PR modulators in the lymph nodes. (I) Migration of the lymphocytes with the lymph to the lymph nodes via afferent lymphatic vessels. (IIa) Binding of the S1PR modulator to the S1PR on the surface of the lymphocyte. (IIIa) Internalisation of the S1PR bound to the modulator. (IVa) Disruption of the migration of the lymphocytes towards the efferent lymphatic vessel. (IIb, IIIb, IVb) Migration of the lymphocytes in the lymph nodes towards the efferent lymphatic vessel following the S1P gradient [77].

Sphingosine 1-phosphate (S1P) is a bioactive lipid molecule playing a crucial role in the regulation of the immune system activity by binding to S1PRs (S1PR1–S1PR5; Figure 3). It was proven that the S1P/S1PR pathway is associated with the development and progression of cancer or immune-mediated diseases. The most recent data show that S1PR modulators and agents inhibiting S1P generation, transport, or degradation may constitute a valuable therapeutic option for the treatment of autoimmune diseases, including IBD [74,75,76]. Until now, two S1PR modulators, ozanimod and etrasimod, have been approved for the treatment of patients with moderate-to-severe UC.

### 5.1. Ozanimod

Ozanimod is the first S1P modulator that has been approved for UC therapy. It binds to S1PR1 and S1PR5, leading to the internalisation of the receptors and a reduction in the number of lymphocytes migrating from the lymph nodes to the intestine. The recommended dose is 0.92 mg, administered orally once daily.

The effectiveness and safety of ozanimod were assessed among 1012 patients with UC aged 18 to 75 in the phase 3, multicentre, randomised, double-blind, placebo-controlled True North trial, conducted from 2015 to 2020. At the end of the induction period, the clinical response to the therapy with ozanimod was observed in 233 out of 429 patients (54.3%). Clinical remission was obtained in 79 (18.4%) patients, compared to 13 (6.0%) of those who received a placebo. At week 52, during the maintenance phase, 138 (60.0%) out of 230 patients receiving ozanimod showed clinical response vs. 93 (41.0%) out of 227 subjects in the placebo arm. Clinical remission was present in 85 (37.0%) and 42 (18.5%) cases, respectively [78]. According to the post hoc analysis, the effectiveness of the treatment was similar between the groups of patients aged <60 and ≥60. However, the researchers noted that the elderly were sparsely represented in the study [79].

During the induction period, 53 subjects aged ≥60 were randomised to double-blind ozanimod (cohort 1), 30 into the placebo arm, and 52 received the open-label ozanimod (cohort 2). In turn, the elderly patients with a clinical response to ozanimod at week 10 were re-randomised to double-blind ozanimod (34 subjects) or the placebo group (31 subjects) for maintenance through 52 weeks. During the induction period, rates of treatment-emergent adverse events with ozanimod in cohorts 1 and 2 were 36% and 31%, respectively, and they were lower than in the <60 years old group. The rates of serious treatment-emergent adverse events were 6% and 4%, and events leading to the discontinuation of treatment were 2% and 6% in cohorts 1 and 2, respectively.

In contrast, during maintenance, the rates of treatment-emergent adverse events, serious treatment-emergent adverse events, and events leading to the discontinuation of therapy were 56%, 3%, and 3%, respectively. The adverse events of special interest were rare, and the most common included infections (2 (5.9%) patients) and macular oedema (1 (2.9%) patient). It must be emphasised that during the induction period, one death from acute respiratory distress syndrome due to viral pneumonia was noticed. However, it was deemed unrelated to ozanimod. In addition, two cases of malignancies were observed in the patients aged <60. In turn, major adverse cardiovascular events, mainly bradycardia in the induction period, were more frequent during ozanimod therapy compared to the placebo and were similar among the older (≥60) and younger (<60) patients. In contrast, liver enzyme abnormalities, another common side effect of therapy, were observed more often in subjects under 20 years old [80,81].

It is worth emphasising that there are no clinically significant differences in the pharmacokinetics of ozanimod connected to age in patients with UC, indicating the lack of need for dose adjustment and relative safety in elderly subjects [82,83]. Noteworthy, there are no clinical studies among elderly patients in the neurology field in which ozanimod was initially approved for the treatment of relapsing multiple sclerosis [84].

### 5.2. Etrasimod

Etrasimod is a S1PR1, S1PR4, and S1PR5 modulator recently approved for the therapy of moderate to severe UC [85]. The recommended dose is 2 mg taken orally once daily. As the introduction of etrasimod to clinical practice happened only a few months ago, the real-life data regarding the safety and effectiveness in all age groups of patients are minimal.

Nonetheless, two independent randomised, multicentre, double-blind, placebo-controlled, phase 3 trials, ELEVATE UC 52 and ELEVATE UC 12, showed that etrasimod was able to induce and maintain remission of moderate to severe UC in patients aged 16 to 80. In ELEVATE UC 52, clinical remission was observed in a majority of patients from the etrasimod group compared with the placebo group after the 12-week induction period (27% vs. 7%; *p* < 0.0001) and at week 52 (32% vs. 7%; *p* < 0.0001). In turn, in ELEVATE UC 12, 25% of patients in the etrasimod group achieved clinical remission, compared with 15% of the patients in the placebo group, at the end of the 12-week induction period (*p* = 0.026).

Side effects were reported in 71% and 47% of the subjects in the etrasimod groups as well as in 56% and 47% of the subjects in the placebo groups in ELEVATE UC 52 and ELEVATE UC 12, respectively. Most commonly, anaemia and headaches were observed, followed by the worsening of UC, dizziness, and COVID-19 infections. In ELEVATE UC 52, 3 (1.0%) serious infections were diagnosed in the etrasimod group and 5 (3.5%) in the placebo arm, with no cases in ELEVATE UC 12. Herpes zoster was reported in 2 (0.7%) subjects from the ozanimod arm in ELEVATE UC 52 and 2 (0.7%) subjects in the placebo arm in ELEVATE UC 12 [86].

As the first-dose transient heart rate reduction and cardiac conduction aberrations are a known side effect of S1P receptor modulators, additional analysis of cardiac abnormalities was conducted by Vermeire et al. [87]. A total of 9 events of bradycardia were reported in the subjects receiving etrasimod in both trials (1.7% of 527 patients), out of which 2 (0.38%) led to therapy discontinuation due to the intensity of symptoms. Moreover, three (0.57%) cases of AV block were reported, where two (0.38%) subjects experienced first-degree AV block and one (0.19%) was diagnosed with second-degree Mobitz type I AV block. Among the subjects affected by the cardiac aberrations, only one patient (0.19%) was aged ≥60.

Moreover, the OASIS study, being a phase 2, randomised, double-blind, placebo-controlled trial, revealed that the daily etrasimod dose of 2 mg was characterised by a favourable safety profile, and most patients achieved clinical response, clinical remission, or endoscopic improvement at week 12 [88]. Nevertheless, there are no studies assessing the effectiveness and safety of etrasimod in elderly patients with UC, resulting in a lack of convincing data about its utility in this patient group. In addition, transient, asymptomatic, low-grade atrioventricular block after the administration of etrasimod was reported in a few patients with moderate to severe UC in a phase 2, double-blind, parallel-group study [89]. This may limit the use of this agent in patients who have cardiac arrhythmia or conduction disturbances, which are frequently observed in elderly subjects.

Of note, according to sub-study A of the CULTIVATE study, evaluating the effectiveness and safety of etrasimod in the therapy of CD (NCT04173273), a phase 2, randomised, double-blind study, with a 14-week induction period followed by a 52-week maintenance phase, including adults aged 18 to 80, endoscopic and clinical improvement was achieved with both etrasimod 2 mg and 3 mg. An endoscopic response was observed in 21.4% and 9.8% of cases, whereas 31.0% and 43.9% of the patients achieved clinical remission, respectively. The incidence of serious adverse events was low (4.8% and 2.4%, respectively). Phase 3 of the study is ongoing [90]. Other investigational oral S1PR modulators, such as prodrugs mocravimod or amiselimod, did not achieve the minimal clinically relevant threshold for effectiveness of remission in the induction phase of the treatment of UC or CD [91,92].

The possible main adverse events and drug interactions, as well as required clinical monitoring during the therapy with the described drugs, are presented in Table 1.

## 6. Conclusions

The number of elderly patients with IBD is constantly increasing. Therefore, new treatment options with a tolerable risk of adverse events are desirable. The emergence of the novel small-molecule drugs has instilled a sense of careful optimism for enhanced therapy outcomes with a potentially acceptable safety profile in this group of subjects. As the novel small-molecule drugs have been approved for the treatment of IBD quite recently, the data are limited, yet encouraging.

Even though the reports concerning the elevated risk of MACE and VTE in patients with RA were published, these findings were not confirmed in different studies regarding the usefulness of JAK inhibitors in the treatment of IBD. The inconsistencies in the results might be related to the heterogeneity of the elderly population, as multiple different comorbidities that may influence the outcome of the therapy are observed among them. As for S1PR modulators, the data are even more scarce, although the outcomes of clinical trials are propitious. In both groups of drugs, the effectiveness in inducing and maintaining the remission of IBD was observed among diverse patients. Moreover, the frequency of adverse events was not elevated in comparison with that in subjects with IBD treated differently.

In conclusion, as the use of thiopurines in senior patients is limited due to the potential adverse events, the introduction of JAK inhibitors and S1PR agonists has provided new promising options in the therapy of IBD for many patients, including the elderly ones. Even so, the sensible selection of eligible subjects is required before the beginning of the treatment, based, among others, on the assessment of the cardiovascular, thromboembolism, and infection risks. Moreover, due to the limited amount of data, further studies, including randomised controlled trials among vast groups of senior subjects, are needed regarding the use of novel small-molecule drugs in the elderly with IBD.

## Figures and Tables

**Table 1:** Table summarising the main characteristics of the discussed drugs that could be used in the therapy of IBD in elderly patients.

Drugs	Main Potential Adverse Events	Drug Interactions	Monitoring
Thiopurines (AZA, 6-MP)	- Increased risk of infections - Leukopenia - Anaemia - Thrombocytopenia - Myelosuppression - Liver injury - Gastric intolerance - Pancreatitis - Increased risk of neoplasms, non-melanoma skin cancer, and lymphoma - Reversible interstitial pneumonia	- Antineoplastic, immunomodulating therapies - Allopurinol, febuxostat - Warfarin - Furosemide - Curare, D-tubocurarine, pancuronium	- Complete blood count, liver enzymes, and bilirubin, creatinine level, and urine test at 2, 4, 6, and 8 weeks after treatment initiation and every 3 months thereafter - Annual TB risk assessment - Annual dermatological skin examination - Infection monitoring during treatment and for up to 3 months after treatment discontinuation
JAK inhibitors	- Increased risk of infections - Suppression of haematopoiesis - Dyslipidaemia - Insomnia - Gastric intolerance - Increased risk of VTE/MACE *	- CYP3A4 substrates, e.g., fluconazole, ketoconazole, tacrolimus, and cyklosporine - Fibrates - Beta-blockers - Calcium channel blockers - Statins - Sartans - Monoclonal antibodies - Steroids	- Complete blood count, liver enzymes, and bilirubin at 4–8 weeks of therapy and every 3 months thereafter - Fasting lipid profile 4–8 weeks after the beginning of the therapy - Annual TB risk assessment - Annual dermatological skin examination - Infection monitoring during treatment and for up to 3 months after treatment discontinuation
S1PR modulators	- Increased risk of infections - Lymphopenia - Bradycardia - Liver injury - Macular oedema - Disturbances of blood pressure	- Antineoplastic, non-corticosteroid immunosuppressive, immunomodulating therapies - Anti-arrhythmic drugs, QT-prolonging drugs - Beta-blockers - Calcium channel blockers - Opioid drugs - Serotonergic/adrenergic drugs - Sympathomimetic - drugs - MAO inhibitors - CYP2C8 inhibitors (clopidogrel, gemfibrozil) - CYP2C8 inducers (rifampin)	- Regular blood pressure monitoring - Infection monitoring during treatment and for up to 3 months after treatment discontinuation - Complete blood count every 3 months - Liver enzymes and bilirubin level at 1, 3, 6, 9, and 12 months after treatment initiation and every 3 months thereafter - Ophthalmic examination if occurring changes in vision or regularly in patients with a history of diabetes, uveitis, or macular oedema

* Data are inconsistent, further research is required. Abbreviations: 6-MP, 6-mercaptopurine; AV, atrioventricular; AZA, azathioprine; EBV, Epstein–Barr virus; ECG, electrocardiogram; JAK, Janus kinase; MACE, major adverse cardiovascular event; MAO, monoamine oxidase; S1PR, Sphingosine 1-phosphate receptor; TB, tuberculosis; TPMT, thiopurine methyltransferase; VTE, venous thromboembolism; VZV, varicella zoster virus [19,82,93].

## Data Availability

The data presented in this study are available upon request from the corresponding author.

## Abbreviations

6-MP	6-mercaptopurine
AZA	azathioprine
CD	Crohn’s disease
IBD	inflammatory bowel disease
JAK	Janus kinase
MACE	major adverse cardiovascular event
NMSC	non-melanoma skin cancer
RA	rheumatoid arthritis
S1PR	Sphingosine 1-phosphate receptor
UC	ulcerative colitis
VTE	venous thromboembolism

## References

[1] Seyedian S.S., Nokhostin F., Malamir M.D. (2019). A review of the diagnosis, prevention, and treatment methods of inflammatory bowel disease. J. Med. Life.

[2] Łodyga M., Eder P., Gawron-Kiszka M., Dobrowolska A., Gonciarz M., Hartleb M., Kłopocka M., Małecka-Wojciesko E., Radwan P., Reguła J. (2021). Guidelines for the management of patients with Crohn’s disease. Recommendations of the Polish Society of Gastroenterology and the Polish National Consultant in Gastroenterology. Gastroenterol. Rev..

[3] Hong S.J., Katz S. (2021). The elderly IBD patient in the modern era: Changing paradigms in risk stratification and therapeutic management. Ther. Adv. Gastroenterol..

[4] Kaplan G.G., Windsor J.W. (2021). The four epidemiological stages in the global evolution of inflammatory bowel disease. Nat. Rev. Gastroenterol. Hepatol..

[5] Clement B., De Felice K., Afzali A. (2023). Indications and safety of newer IBD treatments in the older patient. Curr. Gastroenterol. Rep..

[6] Talar-Wojnarowska R., Caban M., Jastrzębska M., Woźniak M., Strigáč A., Małecka-Wojciesko E. (2024). Inflammatory Bowel Diseases in the Elderly: A Focus on Disease Characteristics and Biological Therapy Patterns. J. Clin. Med..

[7] Hong S.J., Galati J., Katz S. (2022). Crohn’s Disease of the Elderly: Unique Biology and Therapeutic effectiveness and Safety. Gastroenterol. Clin. N. Am..

[8] Khan N., Vallarino C., Lissoos T., Darr U., Luo M. (2017). Risk of Malignancy in a Nationwide Cohort of Elderly Inflammatory Bowel Disease Patients. Drugs Aging.

[9] Khan N., Vallarino C., Lissoos T., Darr U., Luo M. (2020). Risk of Infection and Types of Infection among Elderly Patients with Inflammatory Bowel Disease: A Retrospective Database Analysis. Inflamm. Bowel Dis..

[10] Mosli M.H., Alghamdi M.K., Bokhary O.A., Alzahrani M.A., Takieddin S.Z., Galai T.A., Alsahafi M.A., Saadah O.I. (2023). Inflammatory bowel disease in the elderly: A focus on disease characteristics and treatment patterns. Saudi J. Gastroenterol..

[11] Gargallo-Puyuelo C.J., Laredo V., Gomollón F. (2021). Thiopurines in Inflammatory Bowel Disease. How to Optimize Thiopurines in the Biologic Era?. Front. Med..

[12] Calafat M., Mañosa M., Cañete F., Domènech E. (2021). Clinical Considerations Regarding the Use of Thiopurines in Older Patients with Inflammatory Bowel Disease. Drugs Aging.

[13] Zakerska-Banaszak O., Łykowska-Szuber L., Walczak M., Żuraszek J., Zielińska A., Skrzypczak-Zielińska M. (2022). Cytotoxicity of Thiopurine Drugs in Patients with Inflammatory Bowel Disease. Toxics.

[14] Calafat M., Mañosa M., Cañete F., Ricart E., Iglesias E., Calvo M., Rodríguez-Moranta F., Taxonera C., Nos P., Mesonero F. (2019). Increased risk of thiopurine-related adverse events in elderly patients with IBD. Aliment. Pharmacol. Ther..

[15] Lobatón T., Ferrante M., Rutgeerts P., Ballet V., Van Assche G., Vermeire S. (2015). Effectiveness and safety of anti-TNF therapy in elderly patients with inflammatory bowel disease. Aliment. Pharmacol. Ther..

[16] Cheddani H., Dauchet L., Fumery M., Charpentier C., Marie Bouvier A., Dupas J.L., Pariente B., Peyrin-Biroulet L., Savoye G., Gower-Rousseau C. (2016). Cancer in Elderly Onset Inflammatory Bowel Disease: A Population-Based Study. Am. J. Gastroenterol..

[17] Khan N., Patel D., Trivedi C., Kavani H., Pernes T., Medvedeva E., Lewis J., Xie D., Yang Y.X. (2021). Incidence of Acute Myeloid Leukemia and Myelodysplastic Syndrome in Patients with Inflammatory Bowel Disease and the Impact of Thiopurines on Their Risk. Am. J. Gastroenterol..

[18] Bourrier A., Carrat F., Colombel J.F., Bouvier A.M., Abitbol V., Marteau P., Cosnes J., Simon T., Peyrin-Biroulet L., Beaugerie L. (2016). Excess risk of urinary tract cancers in patients receiving thiopurines for inflammatory bowel disease: A prospective observational cohort study. Aliment. Pharmacol. Ther..

[19] van Gennep S., de Boer N.K., D’Haens G.R., Löwenberg M. (2017). Thiopurine Treatment in Ulcerative Colitis: A Critical Review of the Evidence for Current Clinical Practice. Inflamm. Bowel Dis..

[20] Warner B., Johnston E., Arenas-Hernandez M., Marinaki A., Irving P., Sanderson J. (2018). A practical guide to thiopurine prescribing and monitoring in IBD. Frontline Gastroenterol..

[21] Suárez Ferrer C., González-Lama Y., González-Partida I., Calvo Moya M., Vera Mendoza I., Matallana Royo V., Arevalo Serrano J., Abreu Garcia L. (2019). Usefulness of Thiopurine Monotherapy for Crohn’s Disease in the Era of Biologics: A Long-Term Single-Center Experience. Dig. Dis. Sci..

[22] Jorissen C., Verstockt B., Schils N., Sabino J., Ferrante M., Vermeire S. (2021). Long-term clinical outcome after thiopurine discontinuation in elderly patients with IBD. Scand. J. Gastroenterol..

[23] Rozich J.J., Dulai P.S., Fumery M., Sandborn W.J., Singh S. (2020). Progression of Elderly Onset Inflammatory Bowel Diseases: A Systematic Review and Meta-Analysis of Population-Based Cohort Studies. Clin. Gastroenterol. Hepatol..

[24] Kuenzig M.E., Manuel D.G., Donelle J., Benchimol E.I. (2022). Real world evidence of the association between medication and life expectancy in elderly inflammatory bowel disease: A population-based cohort study. BMC Gastroenterol..

[25] Florin T.H.J., Wright J.D., Jambhrunkar S.D., Henman M.G., Popat A. (2019). A well-tolerated and rapidly acting thiopurine for IBD?. Drug Discov. Today.

[26] Roblin X., Boschetti G., Williet N., Nancey S., Marotte H., Berger A., Phelip J.M., Peyrin-Biroulet L., Colombel J.F., Del Tedesco E. (2017). Azathioprine dose reduction in inflammatory bowel disease patients on combination therapy: An open-label, prospective and randomised clinical trial. Aliment. Pharmacol. Ther..

[27] Alexakis C., Saxena S., Chhaya V., Cecil E., Curcin V., Pollok R. (2017). Do Thiopurines Reduce the Risk of Surgery in Elderly Onset Inflammatory Bowel Disease? A 20-Year National Population-Based Cohort Study. Inflamm. Bowel Dis..

[28] Cheng K., Faye A.S. (2020). Venous thromboembolism in inflammatory bowel disease. World J. Gastroenterol..

[29] Mowat C., Arnott I., Cahill A., Smith M., Ahmad T., Subramanian S., Travis S., Morris J., Hamlin J., Dhar A. (2016). Mercaptopurine versus placebo to prevent recurrence of Crohn’s disease after surgical resection (TOPPIC): A multicentre, double-blind, randomised controlled trial. Lancet Gastroenterol. Hepatol..

[30] Sandborn W.J. (2001). Rational dosing of azathioprine and 6-mercaptopurine. Gut.

[31] Calafat M., Mañosa M., Mesonero F., Guardiola J., Mínguez M., Nos P., Vera I., Taxonera C., Iglesias E., Ricart E. (2020). Switching to a Second Thiopurine in Adult and Elderly Patients with Inflammatory Bowel Disease: A Nationwide Study from the ENEIDA Registry. J. Crohns Colitis.

[32] Lipka S., Vacchio A., Katz S. (2012). P-94 6-Mercaptopurine Side Effect Profile in the Elderly Inflammatory Bowel Disease Patient. Inflamm. Bowel Dis..

[33] Singh A., Mahajan R., Kedia S., Dutta A.K., Anand A., Bernstein C.N., Desai D., Pai C.G., Makharia G., Tevethia H.V. (2022). Use of thiopurines in inflammatory bowel disease: An update. Intest. Res..

[34] Shawky A.M., Almalki F.A., Abdalla A.N., Abdelazeem A.H., Gouda A.M. (2022). A Comprehensive Overview of Globally Approved JAK Inhibitors. Pharmaceutics.

[35] Núñez P., Quera R., Yarur A.J. (2023). Safety of Janus Kinase Inhibitors in Inflammatory Bowel Diseases. Drugs.

[36] Lin C.M., Cooles F.A., Isaacs J.D. (2020). Basic Mechanisms of JAK Inhibition. Mediterr. J. Rheumatol..

[37] Dudek P., Fabisiak A., Zatorski H., Malecka-Wojciesko E., Talar-Wojnarowska R. (2021). Effectiveness, Safety and Future Perspectives of JAK Inhibitors in the IBD Treatment. J. Clin. Med..

[38] O’Shea J.J., Kontzias A., Yamaoka K., Tanaka Y., Laurence A. (2013). Janus kinase inhibitors in autoimmune diseases. Ann. Rheum. Dis..

[39] Ytterberg S.R., Bhatt D.L., Mikuls T.R., Koch G.G., Fleischmann R., Rivas J.L., Germino R., Menon S., Sun Y., Wang C. (2022). Cardiovascular and Cancer Risk with Tofacitinib in Rheumatoid Arthritis. N. Engl. J. Med..

[40] Thorley J. (2021). FDA expands JAK inhibitors warning: Going beyond the data?. Lancet Rheumatol..

[41] FDA Requires Warnings about Increased Risk of Serious Heart-Related Events, Cancer, Blood Clots, and Death for JAK Inhibitors That Treat Certain Chronic Inflammatory Conditions. https://fda.gov/drugs/fda-drug-safety-podcasts/fda-requires-warnings-about-increased-risk-serious-heart-related-events-cancer-blood-clots-and-death.

[42] Li N., Gou Z.P., Du S.Q., Zhu X.H., Lin H., Liang X.F., Wang Y.S., Feng P. (2022). Effect of JAK inhibitors on high- and low-density lipoprotein in patients with rheumatoid arthritis: A systematic review and network meta-analysis. Clin. Rheumatol..

[43] Fries W., Basile G., Bellone F., Costantino G., Viola A. (2023). effectiveness and Safety of Biological Therapies and JAK Inhibitors in Older Patients with Inflammatory Bowel Disease. Cells.

[44] Ericsson S., Eriksson M., Vitols S., Einarsson K., Berglund L., Angelin B. (1991). Influence of age on the metabolism of plasma low density lipoproteins in healthy males. J. Clin. Investig..

[45] Shanmugasundaram M., Rough S.J., Alpert J.S. (2010). Dyslipidemia in the elderly: Should it be treated?. Clin. Cardiol..

[46] Rosada A., Kassner U., Weidemann F., König M., Buchmann N., Steinhagen-Thiessen E., Spira D. (2020). Hyperlipidemias in elderly patients: Results from the Berlin ageing Study II (BASEII), a cross-sectional study. Lipids Health Dis..

[47] Gilroy L., Wilson A. (2024). P783 Exploring the impact of JAK Inhibitors on cholesterol levels in Patients with Inflammatory Bowel Disease: A Real-World Data Analysis. J. Crohns Colitis.

[48] Roda G., Dal Buono A., Argollo M., Danese S. (2020). JAK selectivity: More precision less troubles. Expert. Rev. Gastroenterol. Hepatol..

[49] Olivera P.A., Lasa J.S., Bonovas S., Danese S., Peyrin-Biroulet L. (2020). Safety of Janus Kinase Inhibitors in Patients With Inflammatory Bowel Diseases or Other Immune-mediated Diseases: A Systematic Review and Meta-Analysis. Gastroenterology.

[50] Gilardi D., Gabbiadini R., Allocca M., Correale C., Fiorino G., Furfaro F., Zilli A., Peyrin-Biroulet L., Danese S. (2020). PK, PD, and interactions: The new scenario with JAK inhibitors and S1P receptor modulators, two classes of small molecule drugs, in IBD. Expert Rev. Gastroenterol. Hepatol..

[51] Lichtenstein G.R., Bressler B., Francisconi C., Vermeire S., Lawendy N., Salese L., Sawyerr G., Shi H., Su C., Judd D.T. (2023). Assessment of Safety and effectiveness of Tofacitinib, Stratified by Age, in Patients from the Ulcerative Colitis Clinical Program. Inflamm. Bowel Dis..

[52] Lichtenstein G.R., Bressler B., Vermeire S., Francisconi C., Lawendy N., Shi H., Salese L., Judd D., Loftus E.V. (2020). P539 Assessment of age as a risk factor for adverse events in patients from the tofacitinib ulcerative colitis clinical programme. J. Crohns Colitis.

[53] Khan N., Sundararajan R., Patel M., Trivedi C., Yang Y.X. (2024). Effectiveness of Tofacitinib in Patients with Ulcerative Colitis: A Nationwide Veterans Administration Cohort Study. Am. J. Gastroenterol..

[54] Viola A., Li Voti R., Bivacqua C., De Francesco C., Muscianisi M., Costantino G., Fries W. (2024). Mitigating the Risk of Tofacitinib-induced Adverse Events in the Elderly Population with Ulcerative Colitis. J. Crohns Colitis.

[55] Shehab M., Alrashed F., Alkazemi A., Lakatos P.L., Bessissow T. (2023). Impact of biologic therapies and small molecules on the risk of major adverse cardiovascular events in patients with inflammatory bowel diseases: Systematic review and meta-analysis of randomised controlled trials. Expert Rev. Gastroent..

[56] Sandborn W.J., Ghosh S., Panes J., Vranic I., Wang W., Niezychowski W. (2014). A phase 2 study of Tofacitinib, an oral janus kinase inhibitor, inpatients with crohn’s disease. Clin. Gastroenterol. Hepatol..

[57] Panés J., Sandborn W.J., Schreiber S., Sands B.E., Vermeire S., D’Haens G., Panaccione R., Higgins P.D.R., Colombel J.F., Feagan B.G. (2017). Tofacitinib for induction and maintenance therapy of Crohn’s disease: Results of two phase IIb randomised placebo-controlled trials. Gut.

[58] Dhillon S., Keam S.J. (2020). Filgotinib: First Approval. Drugs.

[59] Turnheim K. (2003). When drug therapy gets old: Pharmacokinetics and pharmacodynamics in the elderly. Exp. Gerontol..

[60] Namour F., Fagard L., Van der Aa A., Harrison P., Xin Y., Tasset C. (2018). Influence of age and renal impairment on the steady state pharmacokinetics of filgotinib, a selective JAK1 inhibitor. Br. J. Clin. Pharmacol..

[61] Schreiber S., Loftus E.V., Maaser C., Danese S., Rudolph C., Jongen R., De Haas A., Oortwijn A., Vermeire S. (2022). DOP37 effectiveness and safety of filgotinib in patients with Ulcerative Colitis stratified by age: Post hoc analysis of the phase 2b/3 SELECTION and SELECTIONLTE studies. J. Crohns Colitis.

[62] Shimada T., Takada J., Baba A., Iwashita M., Hayashi T., Maeda T., Shimizu M. (2024). An Elderly Patient Developed Ulcerative Colitis after SARS-CoV-2 mRNA Vaccination: A Case Report and Review of the Literature. Intern. Med..

[63] Vermeire S., Schreiber S., Petryka R., Kuehbacher T., Hebuterne X., Roblin X., Klopocka M., Goldis A., Wisniewska-Jarosinska M., Baranovsky A. (2017). Clinical remission in patients with moderate-to-severe Crohn’s disease treated with filgotinib (the FITZROY study): Results from a phase 2, double-blind, randomised, placebo-controlled trial. Lancet.

[64] Galapagos Announces Topline Results from Phase 3 DIVERSITY Trial of Filgotinib in Crohn’s Disease. https://www.globenewswire.com/news-release/2023/2/8/2604431/0/en/Galapgos-announces-topline-results-from-Phase-3-DIVERSITY-trial-of-filgotinib-in-Crohn-s-disease.html.

[65] Friedberg S., Choi D., Hunold T., Choi N.K., Garcia N.M., Picker E.A., Cohen N.A., Cohen R.D., Dalal S.R., Pekow J. (2023). Upadacitinib Is Effective and Safe in Both Ulcerative Colitis and Crohn’s Disease: Prospective Real-World Experience. Clin. Gastroenterol. Hepatol..

[66] Trueman S., Mohamed M.F., Feng T., Lacerda A.P., Marbury T., Othman A.A. (2019). Characterization of the Effect of Hepatic Impairment on Upadacitinib Pharmacokinetics. J. Clin. Pharmacol..

[67] Mohamed M.F., Trueman S., Feng T., Anderson J., Marbury T.C., Othman A.A. (2019). Characterization of the Effect of Renal Impairment on Upadacitinib Pharmacokinetics. J. Clin. Pharmacol..

[68] Mohamed M.F., Jungerwirth S., Asatryan A., Jiang P., Othman A.A. (2017). Assessment of effect of CYP3A inhibition, CYP induction, OATP1B inhibition, and high-fat meal on pharmacokinetics of the JAK1 inhibitor upadacitinib. Br. J. Clin. Pharmacol..

[69] Veeravalli V., Dash R.P., Thomas J.A., Babu R.J., Madgula L.M.V., Srinivas N.R. (2020). Critical Assessment of Pharmacokinetic Drug-Drug Interaction Potential of Tofacitinib, Baricitinib and Upadacitinib, the Three Approved Janus Kinase Inhibitors for Rheumatoid Arthritis Treatment. Drug Saf..

[70] Chowla N., Tariq R., Loftus E. (2023). S29 Safety of Upadacitinib in Older Patients with Ulcerative Colitis: A Real-World Experience. Am. J. Gastroenterol..

[71] Levine J., McKibbin J., Ham R., Higgins P., Bishu S., Berinstein J. (2023). Use of upadactinib in 11 tofacitinib-refractory ulcerative colitis patients at a single tertiary care center. Inflamm. Bowel Dis..

[72] Dalal R.S., Kallumkal G., Cabral H.J., Barnes E.L., Allegretti J.R. (2024). One-Year Comparative Effectiveness of Upadacitinib vs Tofacitinib for Ulcerative Colitis: A Multicentre Cohort Study. Am. J. Gastroenterol..

[73] Sandborn W.J., Feagan B.G., Loftus E.V., Peyrin-Biroulet L., Van Assche G., D’Haens G., Schreiber S., Colombel J.F., Lewis J.D., Ghosh S. (2020). Effectiveness and Safety of Upadacitinib in a randomised Trial of Patients with Crohn’s Disease. Gastroenterology.

[74] McGinley M.P., Cohen J.A. (2021). Sphingosine 1-phosphate receptor modulators in multiple sclerosis and other conditions. Lancet.

[75] Pérez-Jeldres T., Alvarez-Lobos M., Rivera-Nieves J. (2021). Targeting Sphingosine-1-Phosphate Signaling in Immune-Mediated Diseases: Beyond Multiple Sclerosis. Drugs.

[76] Bravo G.Á., Cedeño R.R., Casadevall M.P., Ramió-Torrentà L. (2022). Sphingosine-1-Phosphate (S1P) and S1P Signaling Pathway Modulators, from Current Insights to Future Perspectives. Cells.

[77] Bencardino S., D’Amico F., Faggiani I., Bernardi F., Allocca M., Furfaro F., Parigi T.L., Zilli A., Fiorino G., Peyrin-Biroulet L. (2023). Effectiveness and Safety of S1P1 Receptor Modulator Drugs for Patients with Moderate-to-Severe Ulcerative Colitis. J. Clin. Med..

[78] Sandborn W.J., Feagan B.G., Haens G., Wolf D.C., Jovanovic I., Hanauer S.B., Ghosh S., Petersen A., Hua S.Y., Lee J.H. (2021). Ozanimod as Induction and Maintenance Therapy for Ulcerative Colitis. N. Engl. J. Med..

[79] (2022). Post Hoc Analyses of the True North Study Evaluating Ozanimod in Patients with Ulcerative Colitis. Gastroenterol. Hepatol..

[80] Khan N., Irving P., Blumenstein I., Horst S.N., Ahmad H., Lawlor G., Hobbs V., Jain A., Memaj A., Ananthakrishnan A. (2022). S811 Evaluation of ozanimod effectiveness and Safety in Older Patients with Ulcerative Colitis: Post hoc Analysis from the Phase 3 True North Study. Am. J. Gastroenterol..

[81] Sheikh F., Irving P., Ananthakrishnan A., Blumenstein I., Horst S., Ahmad H., Lawlor G., Hobbs V., Jain A., Memaj A. (2023). P100 effectiveness and safety of ozanimod in older patients with ulcerative colitis (UC): Post hoc analysis of the True North study. Gut.

[82] Sands B.E., Schreiber S., Blumenstein I., Chiorean M.V., Ungaro R.C., Rubin D.T. (2023). Clinician’s Guide to Using Ozanimod for the Treatment of Ulcerative Colitis. J. Crohns Colitis.

[83] Shen J., Tatosian D., Sid-Otmane L., Teuscher N., Chen L., Zhang P., Tirucherai G.S., Chitakara D., Marta C. (2021). Population pharmacokinetics of ozanimod and active metabolite cc112273 in patients with ulcerative colitis [abstract p332]. J. Crohns Colitis.

[84] Selmaj K.W., Cohen J.A., Comi G., Bar-Or A., Arnold D.L., Steinman L., Hartung H.P., Montalban X., Havrdova E.K., Cree B.A.C. (2021). Ozanimod in relapsing multiple sclerosis: Pooled safety results from the clinical development program. Mult. Scler. Relat. Disord..

[85] Shirley M. (2024). Etrasimod: First Approval. Drugs.

[86] Sandborn W.J., Vermeire S., Peyrin-Biroulet L., Dubinsky M.C., Panes J., Yarur A., Ritter T., Baert F., Schreiber S., Sloan S. (2023). Etrasimod as induction and maintenance therapy for ulcerative colitis (ELEVATE): Two randomised, double-blind, placebo-controlled, phase 3 studies. Lancet.

[87] Vermeire S., Yarur A., Rubin D.T., Dubinsky M.C., Regueiro M., Irving P., Peyrin-Biroulet L., Goetsch M., Gu G., Wu J. (2023). P476 Characterization of cardiac conduction abnormalities reported in the phase 3 ELEVATE programme. J. Crohns Colitis.

[88] Vermeire S., Chiorean M., Panés J., Peyrin-Biroulet L., Zhang J., Sands B.E., Lazin K., Klassen P., Naik S.U., Cabell C.H. (2021). Long-term Safety and effectiveness of Etrasimod for Ulcerative Colitis: Results from the Open-label Extension of the OASIS Study. J. Crohns Colitis.

[89] Sandborn W.J., Peyrin-Biroulet L., Zhang J., Chiorean M., Vermeire S., Lee S.D., Kühbacher T., Yacyshyn B., Cabell C.H., Naik S.U. (2020). Effectiveness and Safety of Etrasimod in a Phase 2 randomised Trial of Patients with Ulcerative Colitis. Gastroenterology.

[90] D’Haens D., Dubinsky M.C., Peyrin-Biroulet L., Danese S., Sands B.E., Wolf D.C., Yarur A., Chiorean M., Dray D., Modesto I. (2023). P632 Etrasimod induction therapy in moderately to severely active Crohn’s disease: Results from a phase 2, randomised, double-blind substudy. J. Crohns Colitis.

[91] D’Haens G., Danese S., Hibi T., Watanabe M., Davies M. (2019). 1005—A controlled trial of amiselimod, a selective S1P receptor modulator in Crohn’s disease. Gastroenterology.

[92] Radeke H.H., Stein J., Van Assche G., Rogler G., Lakatos P.L., Muellershausen F., Moulin P., Jarvis P., Colin L., Gergely P. (2020). A Multicentre, Double-Blind, Placebo-Controlled, Parallel-Group Study to Evaluate the effectiveness, Safety, and Tolerability of the S1P Receptor Agonist KRP203 in Patients with Moderately Active Refractory Ulcerative Colitis. Inflamm. Intestig. Dis..

[93] Spiewak T.A., Patel A. (2022). User’s guide to JAK inhibitors in inflammatory bowel disease. Curr. Res. Pharmacol. Drug Discov..

